# The influence of socioeconomic status on pre-hospital triage in the Netherlands; a multi-center cohort study

**DOI:** 10.1007/s00068-025-03020-4

**Published:** 2025-12-18

**Authors:** Max Gulickx, Robin D. Lokerman, Job F. Waalwijk, Rogier van der Sluijs, Falco Hietbrink, Mark van Heijl

**Affiliations:** 1 Department of Surgery, University Medical Center Utrecht, Utrecht, The Netherlands; 2 Department of Radiology, University Medical Center Utrecht, Utrecht, The Netherlands; 3 Trauma Center Utrecht, Utrecht, The Netherlands; 4 Department of Surgery, Diakonessenhuis Utrecht/Zeist/Doorn, Utrecht, The Netherlands

**Keywords:** Socioeconomic status, Trauma, Pre-hospital triage, Undertriage, Ambulance

## Abstract

**Purpose:**

Optimizing pre-hospital triage is essential to improve outcomes after severe injury. Sociodemographic factors may influence triage decision, but the role of neighborhood socioeconomic status (SES) at the scene of injury remains unclear. This study evaluated the association between neighborhood SES and the accuracy of pre-hospital trauma triage.

**Methods:**

This multicenter cohort study included all trauma patients transported by eight ambulance regions in the Netherlands between January 2015 and December 2017. Neighborhood SES was defined using income, education, and employment indicators. Outcomes were undertriage and overtriage, based on Injury Severity Score (ISS) ≥ 16. Associations between SES and triage accuracy were analyzed with generalized linear models using inverse probability weighting, adjusted for age, trauma mechanism, injury severity, and trauma center proximity.

**Results:**

A total of 160,109 patients were included, of whom 32,466 (20.2%) were injured in low-SES neighborhoods. Compared with higher-SES areas, these patients were younger (median 55.7 vs. 58.3 years), more often sustained penetrating injuries (1.3% vs. 0.7%), and were injured closer to higher-level centers (median 7.5 vs. 20.3 km). Unadjusted undertriage was lower (14.9% vs. 25.9%) and overtriage higher (27.8% vs. 19.4%). After adjustment, low-SES neighborhoods were associated with increased undertriage risk (aOR 1.30; 95% CI 1.03–1.63) and decreased overtriage risk (aOR 0.65; 95% CI 0.62–0.67).

**Conclusion:**

Neighborhood SES is significantly associated with pre-hospital trauma triage. Severely injured patients in low-SES neighborhoods are at increased risk of undertriage, underscoring the need for targeted strategies to ensure equitable access to specialized trauma care.

**Supplementary Information:**

The online version contains supplementary material available at 10.1007/s00068-025-03020-4.

## Background

Trauma remains a major global cause of death and disability, disproportionately affecting individuals of lower socioeconomic status (SES) [1–3]. Sociodemographic determinants – such as income, education, and neighborhood environment – are well-established predictors of both injury incidence and outcomes [4–7]. Ensuring equitable access to specialized trauma care is therefore critical for reducing avoidable disparities in trauma-related outcomes.

Accurate pre-hospital triage is a critical determinant of inclusive trauma system performance. Undertriage – defined as the transport of a severely injured patient (Injury Severity Score ≥ [ISS] ≥ 16) to a lower-level trauma center – is associated with preventable mortality and long-term disability [8–10]. Conversely, overtriage – the transport of a non-severely injured patient to a higher-level trauma center – strains limited resources and increase healthcare costs [11]. Despite the use of standardized field triage protocols, emerging evidence suggests that triage decisions may be influenced by non-clinical factors. Studies from the United States have demonstrated that Black and Hispanic trauma patients are more likely to be undertriaged than White patients, independent of injury severity of physiological indicators [12]. In addition, geographic and neighborhood-level SES have been linked to reduced access to definitive trauma care, particularly in rural and resource-constrained areas [13, 14]. However, most previous research has focused on individual-level characteristics (e.g., race, insurance status), with limited attention to contextual factors present at the scene of injury, such as neighborhood deprivation, that may influence pre-hospital decision-making.

This study aims to investigate whether sustaining an injury in a low-SES neighborhood is associated with disparities in pre-hospital triage among ambulance transported trauma patients in the Netherlands. The findings may provide valuable insights for refining field triage protocols and promoting equitable access to appropriate trauma care.

## Methods

The current study was reported in accordance with the STROBE guidelines [15]. The Medical.

Ethical Committee of the University Medical Center Utrecht determined that the study did not.

fall under the Medical Research Involving Human Subjects Act (reference number: 20/500747).

### Study setting

This multicenter cohort study was conducted between January 1, 2015, and December 31, 2017, across eight of the 25 ambulance services in the Netherlands, covering seven of the eleven inclusive trauma regions. The participating ambulance services (i.e., Amsterdam-Amstelland, Brabant Midden-West, Brabant-Noord, Gelderland-Zuid, Rotterdam-Rijnmond, Utrecht, Zaanstreek-Waterland, and Zuid-Holland Zuid) collectively transport approximately 550,000 patients annually and serve diverse rural, suburban, and urban areas spanning 8,000km [2], with a population exceeding 6.5 million [16]. Each inclusive trauma region includes one higher-level (level-1) trauma center, which provides specialized trauma care for severely injured patients, and multiple lower-level (level-2 and level-3) trauma centers, which manage mildly and moderately injured patients cost-effectively. Notably, there are no non-trauma centers in the Netherlands and all residents are covered by mandatory universal health insurance.

**Fig. 1:**
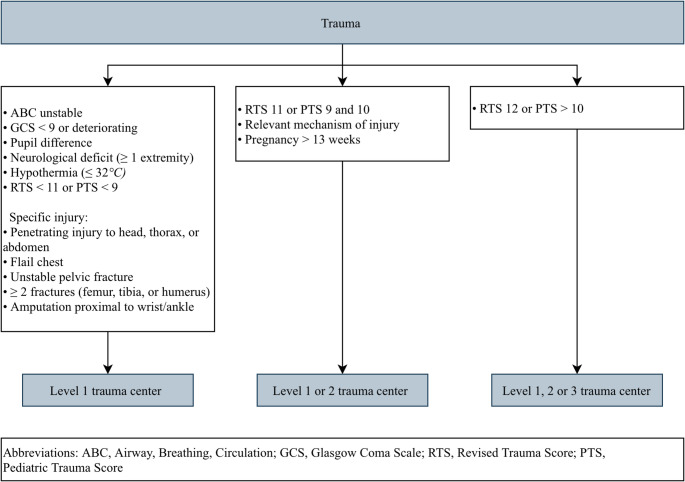
Field triage criteria of the Dutch National protocol of Ambulance Services

In the Netherlands, trauma patients are predominantly transported by ground ambulance; only few patients are transported by helicopter due to the small transportation distances. Ground ambulances are staffed by a specialized nurse trained in advanced trauma life support and a driver who can provide medical assistance. Helicopter Emergency Medical Service (HEMS) teams, consisting of HEMS physician (i.e., trauma surgeon or emergency anesthesiologist), are dispatched to support EMS professionals on scene when a patient is unstable or suspected to be critically injured. Both EMS professionals and HEMS physicians follow the field triage criteria outlined in the National Protocol of Ambulance Services (Fig. 1) [17]. These criteria are derived from the American Field Triage Decision Scheme [18] and help assess the need for specialized trauma care and guide transportation decisions.

### Patients

All trauma patients transported by ground ambulance from one of the eight participating ambulance services to a hospital within the seven participating trauma regions were eligible for inclusion. Patients transported to trauma centers located in non-participating trauma regions were excluded.

### Data collection

Pre-hospital EMS records were linked to hospital records using a unique EMS identifier. For records with missing EMS identifiers, probabilistic linkage was applied using a validated model with 100.0% accuracy, resulting in a complete dataset [19]. Pre-hospital records included data on patient demographics, vital signs, injury mechanism, postal code of the scene of injury, and the transportation destination. Trained data managers of the Dutch Trauma Registry documented injury mechanism, in-hospital diagnosed injuries, and mortality, assigned AIS codes to all admitted patients and calculated the Injury Severity Score (ISS). Patients not admitted to the hospital were assumed not to be severely injured, based on prior evidence demonstrating that all severely injured patients (ISS ≥ 16) are either admitted to the hospital of die in the Emergency Department [20]. 

### Socioeconomic status

Neighborhood socioeconomic status (SES) was determined using the SES-WAO score from the Dutch Central Bureau of Statistics (CBS). This index assigns a status score to each postal code based on financial welfare, education, and employment status. A lower score indicates a lower SES [21]. SES-WAO scores were calculated and linked to the postal codes of the injury location, ranging from − 0.859 to 0.675. Scores were divided into quintiles, with the lowest quintile (status score < −0.259) representing low-SES neighborhoods. The distribution of the SES-WAO scores is shown in Appendix 1. To minimize bias in SES classification, patients injured in postal code areas with fewer than 100 inhabitants were excluded, as such areas may not provide an accurate representation of neighborhood SES.

### Outcomes and definitions

The primary objective of this study was to determine whether sustaining injuries in a low-SES neighborhood was associated with disparities in pre-hospital triage. Undertriage was defined as the transport of a severely injured patient (ISS ≥ 16) to a lower-level trauma center, while overtriage was defined as the transport of a non-severely injured patients (ISS < 16) to a higher-level trauma center. In addition, Early Critical Resource Use (ECRU) was used as a secondary reference standard for accurate triage as previous studies indicate that an ISS greater than 15 – commonly used to determine a patient’s need for transportation to a higher-level trauma center – might not thoroughly correlate with a patient’s need for early critical resources. ECRU was defined as the need for pre-hospital intubation, immediate operative intervention < 24 h (i.e., damage control orthopedics, damage control laparotomy, damage control thoracotomy, ICP monitoring, craniotomy, extremity revascularization, extraperitoneal pelvic packaging), admission to the Intensive Care unit, or death within 24 h [22, 23]. 

### Missing data

Missing data were observed for systolic blood pressure (30.1%), heart rate (19.2%), respiratory rate (37.2%), and Glasgow Coma Scale (14.7%). The missing data appeared to be missing at random (Appendix 2). To address this, multilevel multiple imputation was performed using patient demographics, pre-hospital and hospital vital parameters, injury mechanism, ISS, and mortality. A total of 48 imputed datasets were generated, with 20 iterations per imputation, using the R-package micemd [24]. 

### Statistical analysis

All analyses were conducted using R statistical software (version 4.0.3.) [25]. Descriptive statistics were used to summarize the data. Categorical variables are presented as frequencies with percentages, and continuous variables expressed as medians with interquartile ranges (IQRs). Group differences were assessed using the χ2 test for categorical variables and the Mann–Whitney U test for continuous variables. A p-values of less than 0.05 was considered statistically significant. To evaluate the association between neighborhood-SES and pre-hospital triage, undertriage and overtriage rates were compared between patients from low- and high-SES neighborhoods. Both crude and adjusted odds ratios (ORs) were estimated. Adjusted analyses were performed using generalized linear models (GLMs) with inverse probability weighting (IPW) to account for potential confounding. An additional GLM without inverse probability weighting was performed to test the robustness of our results. As a secondary analysis, SES was modeled as a continuous variable in the IPW-weighted GLM to explore the association between SES and undertriage across the full SES distribution. Covariates included in the models were selected a priori based on clinical relevance and prior literature. These included age, Revised Trauma Score (RTS), ISS, presence of penetrating injury, and driving distance to the nearest higher-level trauma center. Health insurance status was not included as a covariate, as the mandatory coverage in the Netherlands eliminates variability in insurance status. To account for potential non-linearity, restricted cubic splines were applied. To ensure adequate covariate balance, entropy balancing was applied, which iteratively reweights data until balance is achieved [26, 27]. Covariate balance was assessed using the *cobalt* R-package, with Pearson correlation coefficients below 0.1 considered indicative of adequate balance [28]. All statistical analyses were performed in each imputed dataset, and pooled estimates obtained using Rubin’s rules [29, 30]. 

## Results

**Fig. 2:**
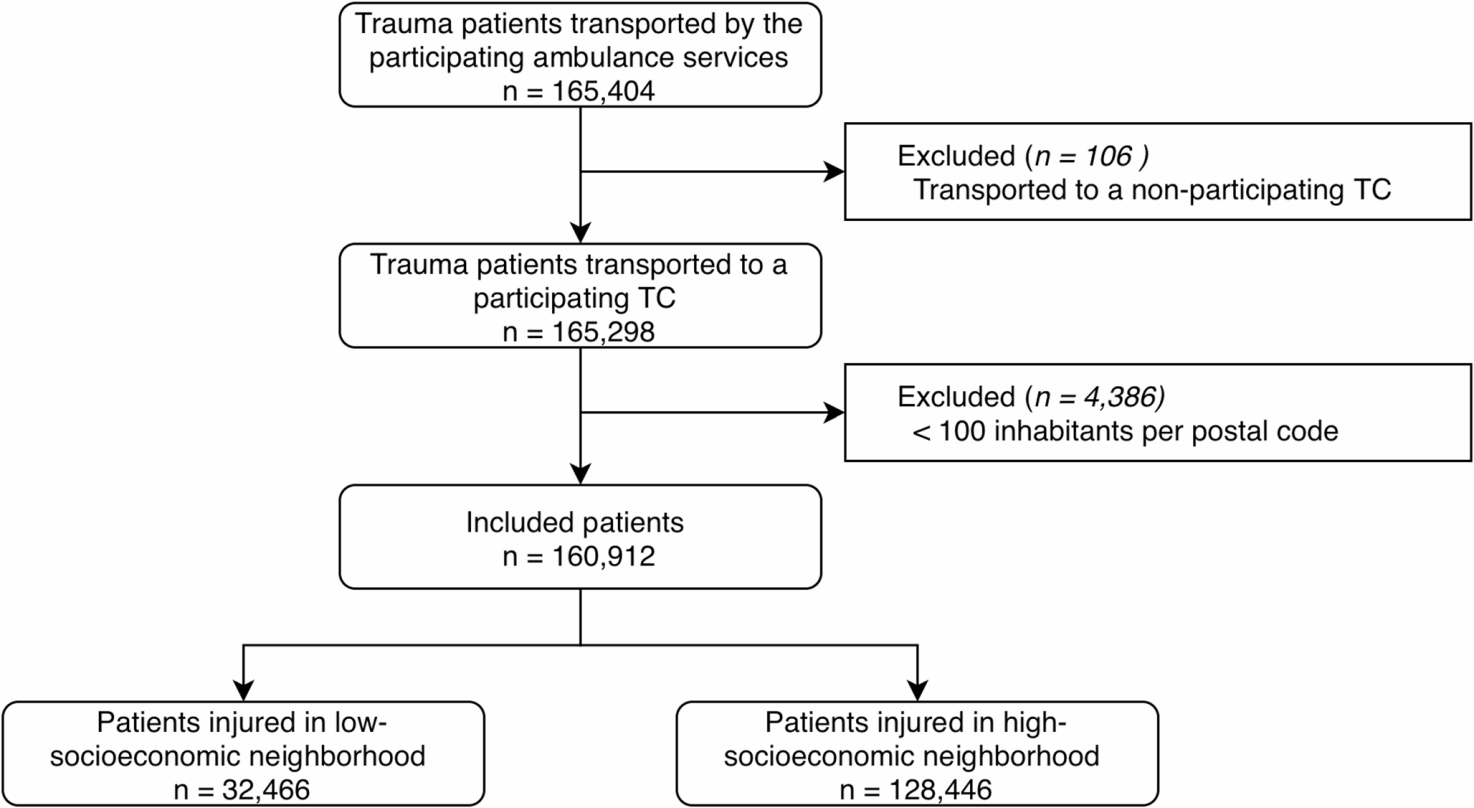
Patient Selection

A total of 165,404 trauma patients were transported by the participating ambulance services during the study period (Fig. 2). After applying inclusion criteria, 160,912 patients were eligible for analysis. The median age was 57.8 years (IQR, 30.4–78.3) and 50.1% of the patients were male. Severe injuries (ISS ≥ 16) were observed in 3,606 (2.2%) patients and a total of 32,466 (20.2%) patients sustained injuries in a low-SES neighborhood (Table 1).

**Table 1:** Baseline characteristics

Variables	Total *n* = 160,912	Low-SES neighborhood *n* = 32,466	High-SES neighborhood *n* = 128,446	***p***value
Demographics	Median (IQR)	Median (IQR)	Median (IQR)	
Age (years)	57.8 (30.4–78.3)	55.7 (29.0–78.1)	58.3 (30.8–78.3)	< 0.001
ISS	6 (4–9)	5 (3–9)	6 (4–9)	< 0.001
	N (%)	N (%)	N (%)	
Age < 16 (years)	12,689 (7.9)	2723 (8.4)	9966 (7.8)	< 0.001
Age ≥ 65 (years)	67,805 (42.1)	13,029 (40.1)	54,776 (42.6)	< 0.001
Male gender	80,604 (50.1)	16,554 (51.0)	64,050 (49.9)	< 0.001
ISS ≥ 16	3606 (2.2)	650 (2.0)	2956 (2.3)	0.001
Mechanism of injury	N (%)	N (%)	N (%)	
High energy trauma	3147 (2.0)	458 (1.4)	2689 (2.1)	< 0.001
Penetrating injury	1348 (0.8)	438 (1.3)	910 (0.7)	< 0.001
Stab or gunshot wound	528 (0.3)	217 (0.7)	311 (0.2)	< 0.001
Vital parameters	N (%)	N (%)	N (%)	
SBP < 90 mmHg	1866 (1.2)	363 (1.1)	1503 (1.2)	0.451
Heart rate > 110 bpm	10,485 (6.5)	2273 (7.0)	8212 (6.4)	< 0.001
Respiratory rate >29/min or < 10/min	3324 (2.1)	757 (2.3)	2567 (2.0)	< 0.001
Glasgow Coma Scale score < 13	5456 (3.4)	1094 (3.4)	4362 (3.4)	0.828
Severe injury (AIS ≥ 3)	N (%)	N (%)	N (%)	
Head and Neck	3968 (2.5)	710 (2.2)	3258 (2.5)	< 0.001
Face	76 (0.0)	27 (0.1)	49 (0.0)	0.001
Thorax	2747 (1.7)	445 (1.4)	2302 (1.8)	< 0.001
Abdomen	472 (0.3)	101 (0.3)	371 (0.3)	0.545
Extremities	15,274 (9.5)	2564 (7.9)	12,710 (9.9)	< 0.001
Pre-hospital characteristics	N (%)	N (%)	N (%)	
HEMS assistance	4173 (2.6)	804 (2.5)	3369 (2.6)	0.143
Highest dispatch priority	81,843(50.9)	17,998 (55.4)	63,845 (49.7)	< 0.001
Transportation characteristics	Median (IQR)	Median (IQR)	Median (IQR)	
Total pre-hospital time, min	41.3 (32.7–51.3)	37.1 (29.8–46.0)	42.5 (33.6–52.5)	< 0.001
Response time, min	8.8 (6–12.3)	7.7 (5.5–10.9)	9.0 (6.2–12.7)	< 0.001
On-scene time, min	18.2 (13–24.7)	18.5 (12.8–25.2)	18.1 (13.0–24.6)	< 0.001
Transport time, min	11.7 (7.3–17.2)	8.7 (5.9–12.7)	12.6 (8.0–18.1)	< 0.001
Distance to high-level TC, km	16.3 (8.0–31.6.0.6)	7.5 (4.6–13.1)	20.3 (10.1–34.3)	< 0.001
	N (%)	N (%)	N (%)	
Initial transportation destination				
Higher-level TC	35,860 (22.3)	9387 (28.9)	26,473 (20.6)	< 0.001
Lower-level TC	125,052 (77.7)	23,079 (71.1)	101,973 (79,4)	< 0.001
Resource use	N (%)	N (%)	N (%)	
Early critical resource use (ECRU)	4240 (2.6)	774 (2.4)	3466 (2.7)	0.002
Emergency intervention < 24 h*	867 (0.5)	200 (0.6)	667 (0.5)	0.037
Pre-hospital intubation	1608 (1.0)	283 (0.9)	1325 (1.0)	0.011
ICU-admission	2710 (1.7)	470 (1.4)	2240 (1.7)	< 0.001
24 h mortality	323 (0.2)	57 (0.2)	266 (0.2)	0.287

Abbreviations:* SES* Socioeconomic Status, *ISS* Injury Severity Score, *SBP* systolic blood pressure, *ICU* Intensive Care Unit, *IQR* Interquartile range. SBP was missed in 30.1%, heart rate in 19.2%, respiratory rate in 37.2%, and Glasgow Coma Scale in 14.7%. *Emergency intervention: Damage control orthopedics, Damage control laparotomy, Damage control thoracotomy, ICP monitoring, Craniotomy, Extremity revascularization, Extraperitoneal pelvic packaging

### Impact of Low-SES neighborhood on patient characteristics and triage

Patients injured in a low-SES neighborhood were significantly younger than those injured in high-SES areas (median age [IQR], 55.7 [29.0–78.1] vs. 58.3 [30.8–78.3]; *p* < 0.001). Although the prevalence of severe injury was slightly less common in these patients (2.0% vs. 2.3%, *p* = 0.001), penetrating injuries occurred more frequently (1.3% vs. 0.7%; *p* < 0.001). Ambulances dispatches for patients in low-SES neighborhoods were more often assigned the highest priority (47.7% vs. 40.9%; *p* < 0.001). Moreover, these patients were more frequently transported to a higher-level trauma center (28.9% vs. 20.6%; *p* < 0.001), coinciding with shorter median driving distances to the nearest higher-level trauma center (7.5 km [IQR, 4.6–13.1] vs. 20.3 km [IQR, 10.1–34.3]; *p* < 0.001). Baseline characteristics per SES quintile are presented in Appendix 3.

### Triage accuracy by neighborhood SES

Triage performance metrics are presented in Table 2. Among patients with severe injuries, undertriage occurred less frequently in low-SES neighborhoods compared to high-SES areas (14.9% vs. 25.9%). Additionally, among non-severely injured patients, overtriage was more common in low-SES neighborhoods (27.8% vs. 19.4%). In unadjusted analyses, low-SES injury location was associated with lower odds of undertriage (OR 0.50 [95% CI; 0.40–0.63]) and a higher odds of overtriage (OR 1.60 [95% CI; 1.56–1.65]). After adjustment for demographics, injury severity and mechanism, and trauma center proximity, sustaining injuries in a low-SES neighborhood was independently associated with increased odds of undertriage (OR 1.30 [95% CI; 1.03–1.63]) and decreased odds of overtriage (OR 0.65 [95% CI; 0.62–0.67]). An additional GLM without the use of IPW was performed to assess the robustness of these findings and yielded similar results, with an adjusted OR of 1.27 (95% CI, 1.01–1.69) for undertriage and 0.70 (95% CI, 0.67–0.72) for overtriage. Secondary analysis indicated that each standard deviation decrease in SES score was associated with a 27% higher odds of undertriage (aOR 1.27; 95% CI, 1.14–1.41).

**Table 2:** Pre-hospital triage in low and high-socioeconomic neighborhoods

Pre-hospital triage	No. %		Crude analysis	Adjusted analysis GLM fit with IPW^a^	Adjusted analysis GLM^a^
	Low-SES	High-SES	Estimate (95%-CI)	Estimate (95%-CI)	Estimate (95%-CI)
Patient ISS, No					
≥ 16	650	2956	NA	NA	NA
< 16	31,816	125,490	NA	NA	NA
Undertriage^b^	97 (14.9)	767 (25.9)	0.50 (0.40–0.63)	1.30 (1.03–1.63)	1.27 (1.01–1.69)
Overtriage^c^	8834 (27.8)	24284 (19.4)	1.60 (1.56–1.65)	0.65 (0.62–0.67)	0.70 (0.67–0.72)
Resource use, No					
ECRU	774	3466	NA	NA	NA
No ECRU	31,692	124,980	NA	NA	NA
ECRU undertriage^d^	186 (24.0)	1023 (29.5)	0.76 (0.63–0.90)	1.25 (1.02–1.54)	1.51 (1.19–1.91)
ECRU overtriage^e^	8799 (27.8)	24030 (19.2)	1.61 (1.57–1.66)	0.60 (0.57–0.64)	0.65 (0.61–0.69)
Continuous SES analysis^f^					
ISS undertriage	NA	NA	0.84 (0.83–0.85)	1.27 (1.14–1.41)	1.20 (1.18–1.22)
ECRU undertriage	NA	NA	0.97 (0.95–0.99)	1.30 (1.19–1.43)	1.25 (1.22–1.28)

*GLM*: Generalized Linear Model, *IPW*: Inverse Probability Weighting, *ISS*: Injury Severity Score, *ECRU*: Early Critical Resource Use, *NA*: Not Applicable

^a^High-socioeconomic neighborhoods served as a reference standard. Adjusted for: gender, Revised Trauma Score (RTS), Injury Severity Score (ISS), penetrating injury, and distance to closest higher-level trauma center

^b^Undertriage was defined as a severely injured patient (ISS ≥ 16) transported to a lower-level trauma center

^c^Overtriage was defined as a non-severely injured patient (ISS <16) transported to a higher-level trauma center

^d^ECRU undertriage was defined as a patient in need of early critical resources and transported to a lower-level trauma center

^e^ECRU overtriage was defined as a patient not in need of early critical resources and transported to a higher-level trauma center

^f^Per Standard Deviation decreas in neighborhood SES score

### Sensitivity analysis using early critical resource use as triage reference standard

Consistent findings were obtained when ECRU was employed as an alternative triage reference standard. After adjustment, patients from low-SES neighborhoods who required ERCU had increased odds of undertriage (adjusted OR [95%-CI], 1.25 [1.02–1.54]) and decreased odds of overtriage (adjusted OR [95%-CI], 0.60 [0.57–0.64]). Secondary analysis indicated that each standard deviation decrease in SES score was associated with a 30% increased odds of undertriage (aOR 1.30; 95% CI, 1.19–1.43).

## Discussion

This multicenter cohort study is the first, to our knowledge, to examine the association between neighborhood-level SES and pre-hospital trauma triage in the Netherlands. We found that patients injured in low-SES neighborhoods were generally located closer to higher-level trauma centers and were more frequently transported to such centers. Crude rates reflected this proximity: undertriage rates were lower, and overtriage rates were higher in low-SES neighborhoods compared to higher-SES areas. However, after adjustment for demographic characteristics, injury severity and mechanism, vital signs, and trauma center proximity, sustaining injuries in low-SES neighborhoods was independently associated with increased odds of undertriage (adjusted OR 1.30 [1.03–1.63]) and decreased odds of overtriage (adjusted OR 0.65 [0.62–0.67]). These associations remained consistent when ECRU was used as an alternative triage reference standard and were robust across sensitivity analyses.

In the Netherlands, low-SES neighborhoods are predominantly situated in urban areas with relatively short traveling distances to higher-level trauma centers. Prior work by Waalwijk et al. has demonstrated a strong inverse relationship between distance to a trauma center and triage accuracy, with undertriage likelihood increasing by 83% for every 10-kilometer increment in distance [31]. Accordingly, the geographic proximity of low-SES neighborhoods initially suggests an advantage in access to specialized trauma care. This proximity, combined with the observed higher incidence of penetrating injuries and more frequent assignment of the higher dispatch priority, likely contributed to the higher transport rates to higher-level trauma centers and the superficially lower undertriage rates observed in crude analyses. Similar trends have been reported in U.S. based studies by Alber et al. where Black and Hispanic patients – who often reside in urban neighborhoods near trauma centers – exhibited lower crude undertriage rates but were at risk for undertriage after adjusted analyses (Black patients [crude OR 0.73, adjusted OR 1.20]; Hispanic patients [crude OR 0.92, adjusted OR 1.39]) [12]. 

Although factors like trauma-center proximity and penetrating injury patterns may have partially reduced undertriage rates in low-SES neighborhoods, adjusted analyses controlling for key clinical and geographic variables revealed underlaying disparities in triage accuracy. This indicates that access and clinical presentation alone do not fully explain the differences in pre-hospital triage, suggesting that additional, less visible determinants may influence pre-hospital decision-making in socioeconomically disadvantaged environments. Several mechanisms may underlie these disparities. First, patients in socioeconomically disadvantaged areas may present with higher rates of chronic comorbidities – such as diabetes, cardiovascular disease, or substance use – that can obscure physiological markers of trauma severity, complicating pre-hospital injury assessment leading to underrecognition of injury severity. Second, environmental characteristics of low-SES neighborhoods – such as high-density housing, chaotic or unsafe scenes, and language barriers – can impede effective scene management and may influence triage decisions. Third, cognitive heuristics and implicit biases may lead – unintentionally – to underestimation of injury severity based on social context or environmental influence on the scene. Together, these factors may impede accurate pre-hospital triage and help explain the association observed between sustaining injuries in low-SES neighborhoods and undertriage. Addressing these disparities is crucial, as timely and appropriate triage is fundamental to improving trauma outcomes and ensuring equitable access to specialized trauma care.

### Strengths and limitations

This study has several strengths. First, it encompasses a large, multicenter cohort representing nearly one-third of all Dutch ambulance services and two-thirds of the national inclusive trauma systems, thereby enhancing generalizability across diverse geographic and sociodemographic settings. The large sample size also provided sufficient power to detect associations and perform subgroup analyses. However, such large numbers may render small absolute differences statistically significant without reflecting clinically meaningful disparities (e.g., minor baseline variations; Table 1). These differences should therefore be interpreted considering their clinical relevance when comparing groups. Second, methodologically, advanced statistical techniques were applied, including multiple imputation to address missing data and entropy balancing to ensure covariate balance. Sensitivity analyses using alternative modeling approaches and outcome definitions (i.e., Early Critical Resource Use) yielded consistent results, supporting the validity of our results.

However, several limitations should also be acknowledged. First, individual-level SES data were unavailable due to anonymization of the data. Although neighborhood SES is a relevant proxy for pre-hospital decision making due to scene-of-injury influences, it cannot fully account for individual socioeconomic heterogeneity. Second, missing vital parameters were addressed using multiple imputation. This approach preserved the full cohort and minimized information loss but relies on assumptions about the relationship between observed and missing data. As imputed values may not fully reflect the true measurements, some residual bias cannot be excluded. Third, although ISS ≥ 16 is the reference standard for trauma triage evaluation, it does not always reflect real-time clinical judgement or need for urgent interventions. To address this, ECRU was included as a complementary outcome measure.

Although trauma care is ought to be guided primarily by clinical need, our findings demonstrate that disparities in pre-hospital triage exist based on the SES of the scene of injury. Severely injured patients from low-SES neighborhoods are at increased risk of undertriage, potentially withholding access to specialized trauma care and increasing the risk of preventable mortality and disability. Addressing these disparities will require continued efforts within inclusive trauma systems, including raising awareness among EMS professionals, incorporating socioeconomic factors into triage evaluation, and facilitating the clinical implementation of support tools to aid EMS professionals during triage decisions. Future research should further explore the role of socioeconomic context in pre-hospital triage and incorporate these findings into the optimization of triage decision-support strategies.

## Conclusion

In this multicenter cohort study, sustaining injuries in low-SES neighborhoods was independently associated with pre-hospital triage, with severely injured patients facing an increased risk of undertriage. Our findings suggest that socioeconomic factors of the scene of injury may influence pre-hospital decision making. Future research is needed to better understand the mechanisms underlaying these disparities and to identify strategies to reduce undertriage and ensure equity in access to adequate trauma care.

## Supplementary Information

Below is the link to the electronic supplementary material.

Supplementary Material 1 (DOCX. 23.6 KB)

Supplementary Material 2 (DOCX. 18.3 KB)

Supplementary Material 3 (DOCX. 20.6 KB)

## Data Availability

The data that supports the findings of the current study is not publicly available due to its sensitive nature but is available upon a reasonable request that needs to be approved by the participating Emergency Medical Services and trauma regions, provided that appropriate ethical approval is sought. R-scripts are available upon request.
