# Supplementary figures and images for: The influence of socioeconomic status on pre-hospital triage in the Netherlands; a multi-center cohort study
Source: Eur J Trauma Emerg Surg. 2025 Dec 18;51(1):365. doi: 10.1007/s00068-025-03020-4 (PMC12714789; doi:10.1007/s00068-025-03020-4)

**Appendix**


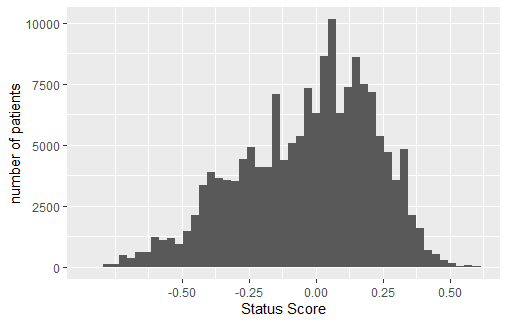


Appendix 1. Socioeconomic status score distribution

Supplement: Supplementary file 1 — Supplementary Material 1 (DOCX. 23.6 KB) [file 68_2025_3020_MOESM1_ESM.docx]
