# Supplementary material for: The influence of socioeconomic status on pre-hospital triage in the Netherlands; a multi-center cohort study
Source: Eur J Trauma Emerg Surg. 2025 Dec 18;51(1):365. doi: 10.1007/s00068-025-03020-4 (PMC12714789; doi:10.1007/s00068-025-03020-4)
Supplement: Supplementary file 2 — Supplementary Material 2 (DOCX. 18.3 KB) [file 68_2025_3020_MOESM2_ESM.docx]

| **Appendix 2.** Baseline characteristics divided in patients with and wihout a missing vital parameter | | | |
| --- | --- | --- | --- |
| **Variables** | Total  n = 160,912 | Patients with a missing vital parameter  n = 79,163 | Patients without a missing vital parameter  n = 81,749 |
| **Demographics** | **Median (IQR)** | **Median (IQR)** | **Median (IQR)** |
| Age (years) | 57.8 (30.4 – 78.3) | 55.6 (26.5 – 78.0) | 59.6 (34.6 – 78.6) |
| ISS | 6 (4-9) | 5 (4-9) | 8 (4-9) |
|  | **N (%)** | **N (%)** | **N (%)** |
| Age <16 (years) | 12689 (7.9) | 9499 (12.0) | 3190 (3.9) |
| Age ≥ 65 (years) | 67805 (42.1) | 32077 (40.5) | 35705 (43.7) |
| Male gender | 80604 (50.1) | 39164 (49.5) | 41437 (50.7) |
| ISS ≥16 | 3606 (2.2) | 1395 (1.8%) | 2211 (2.7) |
| Early critical resource use (ECRU) | 4240 (2.6) | 1728 (2.2) | 2511 (3.1) |
| Low SES Neighborhood | 32,462 (20.3) | 17575 (22.2) | 14887 (18.2) |
| **Mechanism of injury** | **N (%)** | **N (%)** | **N (%)** |
| High energy trauma | 3147 (2.0) | 1355 (1.7) | 1792 (2.2) |
| Penetrating injury | 1348 (0.8) | 698 (0.9) | 650 (0.8) |
| **Vital parameters** | **N (%)** | **N (%)** | **N (%)** |
| SBP <90 mmHg | 1866 (1.2) | 409 (0.5) | 1271 (1.6) |
| Heart rate >110 bpm | 10,485 (6.5) | 4080 (5.2) | 6404 (7.8) |
| Respiratory rate  > 29/min or  < 10/min | 3324 (2.1) | 955 (1.2) | 2124 (2.6) |
| Glasgow Coma Scale score < 13 | 5456 (3.4) | 1777 (2.2) | 3667 (4.5) |
| **Transportation characteristics** | **Median (IQR)** | **Median (IQR)** | **Median (IQR)** |
| Distance to high-level TC, km | 16.3 (8.0-31.6) | 14.9 (7.4 – 30.2) | 17.7 (8.7 – 33.0) |
| Initial transportation destination |  |  |  |
| Higher-level TC | 35,860 (22.3) | 16143 (20.4) | 19715 (24.1) |
| Lower-level TC | 125,052 (77.7) | 63009 (79.6) | 62031 (75.9) |
| HEMS assistance | 4173 (2.6) | 1451 (1.8) | 2618 (3.2) |
| **Outcome** | **N (%)** | **N (%)** | **N (%)** |
| 24 h mortality | 323 (0.2) | 165 (0.2) | 158 (0.2) |
| Abbreviations: SES; Socioeconomic status, SBP; systolic blood pressure, TC; trauma center, HEMS; helicopter emergency medical service | | | |

Appendix 2.
