# Supplementary material for: The influence of socioeconomic status on pre-hospital triage in the Netherlands; a multi-center cohort study
Source: Eur J Trauma Emerg Surg. 2025 Dec 18;51(1):365. doi: 10.1007/s00068-025-03020-4 (PMC12714789; doi:10.1007/s00068-025-03020-4)
Supplement: Supplementary file 3 — Supplementary Material 3 (DOCX. 20.6 KB) [file 68_2025_3020_MOESM3_ESM.docx]

| **Appendix 3.** Baseline characteristics per SES score quinitiles | | | | | | |
| --- | --- | --- | --- | --- | --- | --- |
| **Variables** | Total  n = 160,912 | SES Lowest  n = 32,466 | SES 2  n = 31,954 | SES 3  n = 32,493 | SES 4  n = 32,160 | SES Highest  n = 31,839 |
| **Demographics** | **Median (IQR)** | **Median (IQR)** | **Median (IQR)** | **Median (IQR)** | **Median (IQR)** | **Median (IQR)** |
| Age (years) | 57.8 (30.4 – 78.3) | 55.7 (29.0 – 78.1) | 57.8 (30.8 – 78.9) | 59.0 (31.2 – 78.7) | 59.6 (31.9 – 78.7) | 56.7 (29.2 – 76.7) |
| ISS | 6 (4-9) | 5 (3 – 9) | 6 (4 – 9) | 8 (4 – 9) | 8 (4 – 9) | 8 (4 – 9) |
|  | **N (%)** | **N (%)** | **N (%)** | **N (%)** | **N (%)** | **N (%)** |
| Age <16 (years) | 12689 (7.9) | 2723 (8.4) | 2180 (6.8) | 2238 (6.9) | 2468 (7.7) | 3080 (9.7) |
| Age ≥ 65 (years) | 67805 (42.1) | 13025 (40.1) | 13600 (42.6) | 14099 (43.4) | 14084 (43.8) | 12974 (40.8) |
| Male gender | 80604 (50.1) | 16554 (51.0) | 15798 (49.4) | 16163 (49.7) | 15960 (49.6) | 16126 (50.7) |
| ISS ≥16 | 3606 (2.2) | 650 (2.0) | 603 (1.9) | 682 (2.1) | 769 (2.4) | 902 (2.8) |
| Early critical resource use (ECRU) | 4240 (2.6) | 774 (2.4) | 768 (2.4) | 772 (2.4) | 895 (2.8) | 1030 (3.2) |
| **Mechanism of injury** | **N (%)** | **N (%)** | **N (%)** | **N (%)** | **N (%)** | **N (%)** |
| High energy trauma | 3147 (2.0) | 438 (1.3) | 446 (1.4) | 519 (1.6) | 717 (2.2) | 1007 (3.2) |
| Penetrating injury | 1348 (0.8) | 458 (1.4) | 648 (2.0) | 212 (0.7) | 219 (0.7) | 187 (0.6) |
| **Vital parameters** | **N (%)** | **N (%)** | **N (%)** | **N (%)** | **N (%)** | **N (%)** |
| SBP <90 mmHg | 1866 (1.2) | 316 (1.0) | 316 (1.0) | 360 (1.1) | 346 (1.1) | 342 (1.1) |
| Heart rate >110 bpm | 10,485 (6.5) | 2273 (7.0) | 2106 (6.6) | 2101 (6.5) | 2070 (6.4) | 1934 (6.1) |
| Respiratory rate  > 29/min or  < 10/min | 3324 (2.1) | 699 (2.2) | 593 (1.9) | 571 (1.8) | 585 (1.8) | 631 (2.0) |
| Glasgow Coma Scale score < 13 | 5456 (3.4) | 1093 (3.4) | 1135 (3.6) | 1154 (3.6) | 1052 (3.3) | 1010 (3.2) |
| **Transportation characteristics** | **Median (IQR)** | **Median (IQR)** | **Median (IQR)** | **Median (IQR)** | **Median (IQR)** | **Median (IQR)** |
| Distance to high-level TC, km | 16.3 (8.0-31.6) | 7.5 (4.6 – 13.1) | 14.3 (8.3 – 29.6) | 19.2 (9.5 – 32.8) | 28.5 (13.1 – 38.6) | 21.9 (12.5 – 34.0) |
| Initial transportation destination |  |  |  |  |  |  |
| Higher-level TC | 35,860 (22.3) | 9387 (28.9) | 6111 (19.1) | 7144 (22.0) | 5584 (17.4) | 7632 (24.0) |
| Lower-level TC | 125,052 (77.7) | 23076 (71.1) | 25841 (80.9) | 25346 (78.0) | 26573 (82.6) | 24204 (76.0) |
| HEMS assistance | 4173 (2.6) | 795 (2.4) | 648 (2.0) | 798 (0.5) | 873 (2.7) | 955 (3.0) |
| **Outcome** | **N (%)** | **N (%)** | **N (%)** | **N (%)** | **N (%)** | **N (%)** |
| 24 h mortality | 323 (0.2) | 57 (0.2) | 62 (0.2) | 53 (0.2) | 69 (0.2) | 82 (0.3) |
| Abbreviations: SES; Socioeconomic status, SBP; systolic blood pressure, TC; trauma center, HEMS; helicopter emergency medical service | | | | | | |

Appendix 3.
